# Tissue‐derived extracellular vesicle profiling identifies GLUT1 enabling ultrasensitive circulating quantification and early detection of non‐small cell lung cancer

**DOI:** 10.1002/ctm2.70647

**Published:** 2026-04-08

**Authors:** Heng Huang, Taketo Kato, Yuichi Abe, Akira Yokoi, Masami Kitagawa, Eri Asano‐Inami, Taiki Ryo, Yoshito Imamura, Yuji Nomata, Hirofumi Takenaka, Hiroki Watanabe, Yuta Kawasumi, Keita Nakanishi, Yuka Kadomatsu, Harushi Ueno, Shota Nakamura, Tetsuya Mizuno, Ayumu Taguchi, Toyofumi Fengshi Chen‐Yoshikawa

**Affiliations:** ^1^ Department of Thoracic Surgery Nagoya University Graduate School of Medicine Nagoya Japan; ^2^ Immunoproteomics Laboratory, Institute for Glyco‐core Research (iGCORE) Gifu University Gifu Japan; ^3^ Department of Obstetrics and Gynecology Nagoya University Graduate School of Medicine Nagoya Japan; ^4^ Department of Molecular Oncology Nagoya City University Graduate School of Medical Sciences Nagoya Japan

1

Dear Editor,

Extracellular vesicle (EV)‐based liquid biopsy is promising for early detection of non‐small cell lung cancer (NSCLC), but hindered by the low abundance and limited cancer specificity of circulating EV cargoes. Using multi‐omics integration, we identified glucose transporter 1 (GLUT1) as a membrane‐localised biomarker in tissue‐derived EVs and achieved ultrasensitive circulating quantification with plasma samples, potentially discriminating NSCLC from benign lesions, including in computed tomography (CT)‐suspected indeterminate pulmonary nodules (IPNs).

EVs from body fluids or cell lines have been well studied, whereas tissue‐derived EVs may offer greater cancer specificity by concentrating tumour‐proximal secretomes and minimising systemic contamination, thereby providing more representative biological insights.^1^, ^2^ In our study, tissue‐derived EVs were isolated by ultracentrifugation with density gradient flotation and subjected to proteomic profiling (Figure 1). Tissue‐derived EVs exhibited comparable characteristics to cell line‐derived EVs, and demonstrated distinct protein profiles between cancer and non‐cancer groups (Figure 2A–D). A total of 904 differentially expressed proteins (DEPs) were initially screened (NUH group; Figure 2E–G). Enrichment analyses linked the top‐ranked DEPs to microenvironment‐related processes and glycolysis/gluconeogenesis, facilitating subsequent biologically grounded biomarker selection (Figure 2H and I).

**FIGURE 1:**
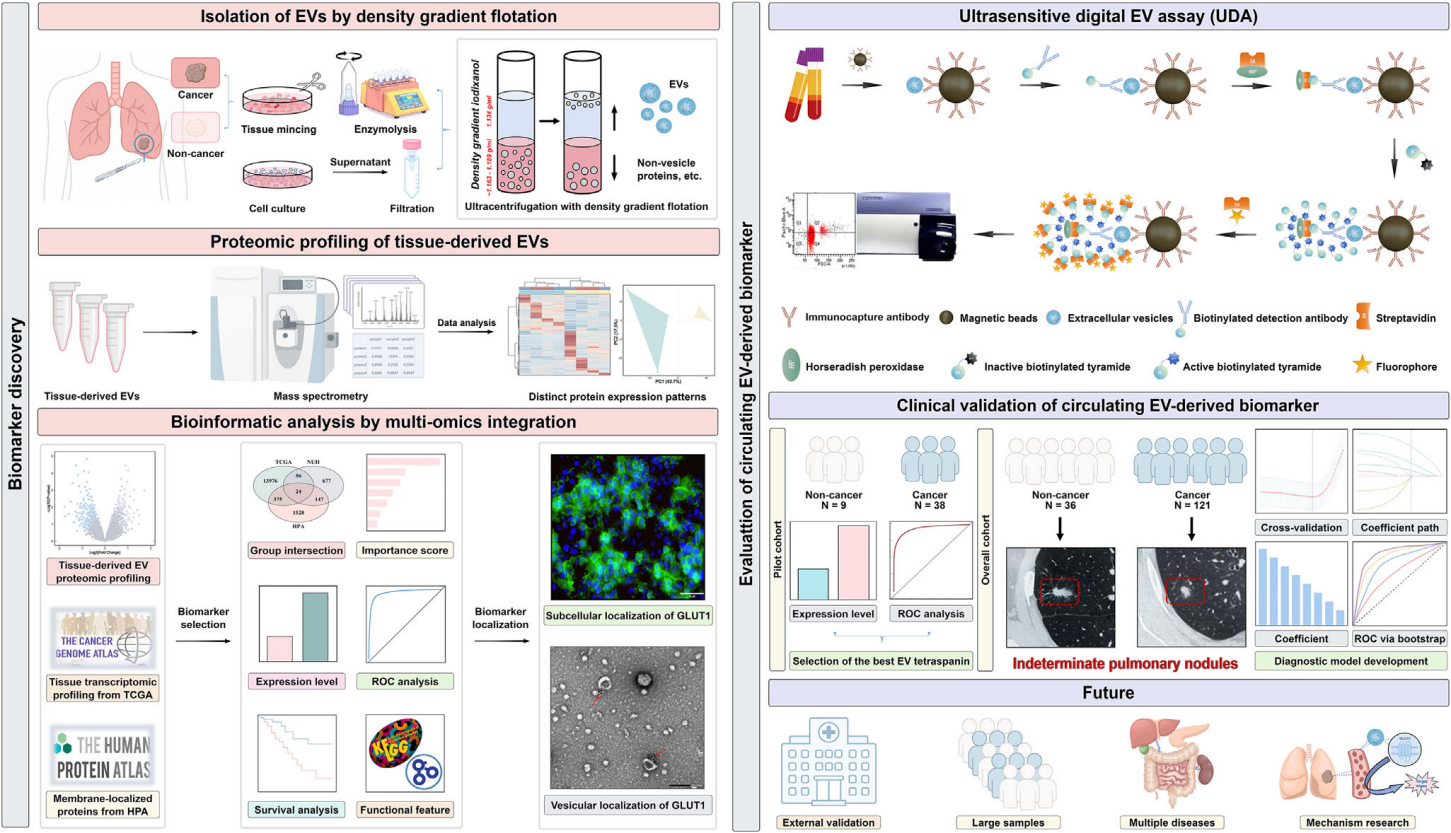
Integrative workflow for tissue‐derived EV biomarker discovery in early detection of NSCLC. EV, extracellular vesicle; GLUT1, glucose transporter 1; HPA, Human Protein Atlas; NSCLC, non‐small cell lung cancer; NUH, Nagoya University Hospital; ROC, receiver operating characteristic; TCGA, The Cancer Genome Atlas; UDA, ultrasensitive digital EV assay.

**FIGURE 2:**
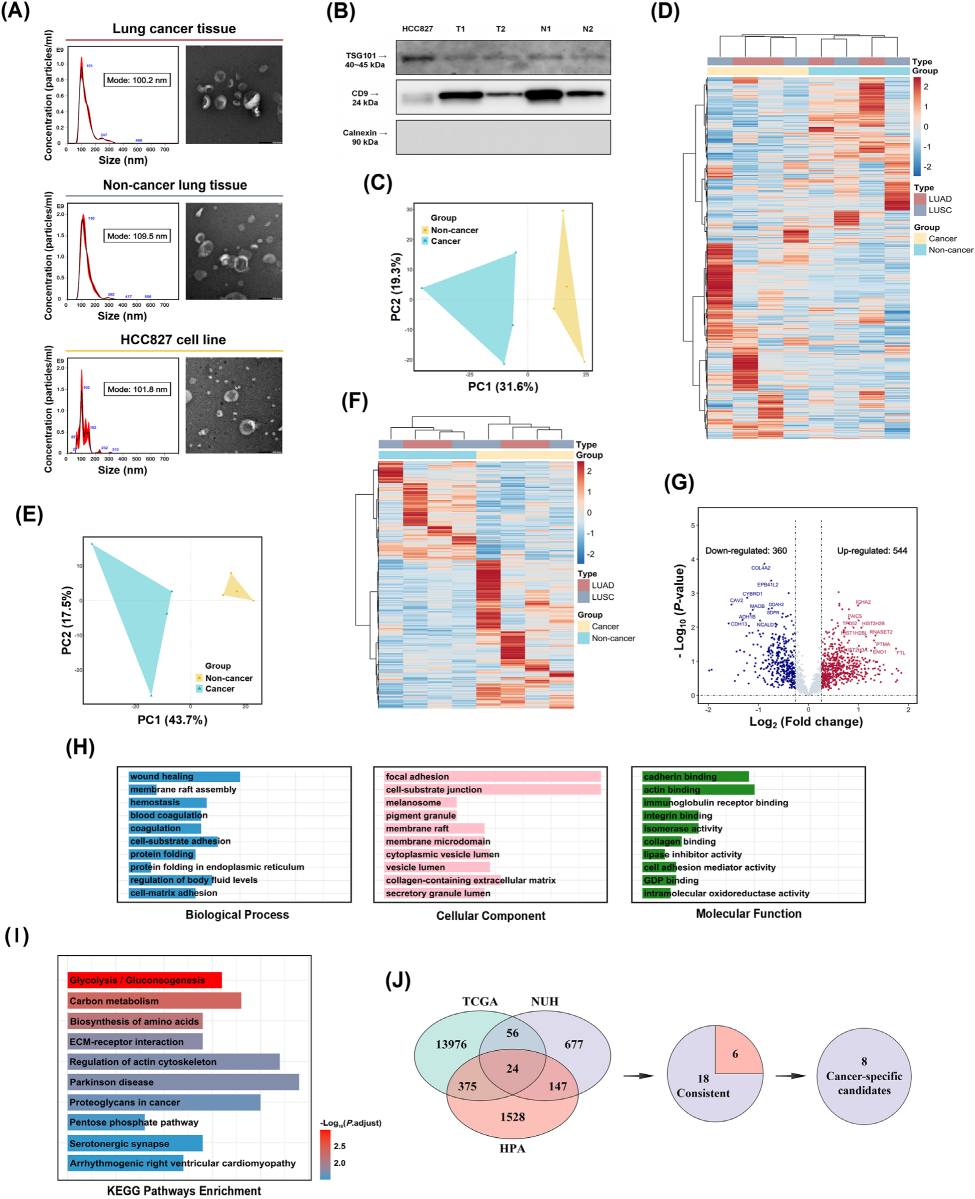
Tissue‐derived EV isolation and proteomic profiling. (A) NTA‐based EV characterisation with TEM images and (B) WB analyses of TSG101 and CD9 (EV markers), and calnexin (cellular contamination marker) on the same representative tissue samples and the HCC827 cell line. (C) PCA plot and (D) hierarchical clustering heatmap of all proteomic profiles of tissue‐derived EVs. (E) PCA plot, and (F) hierarchical clustering heatmap of DEPs of tissue‐derived EVs. (G) Volcano plot visualising the distribution of DEPs. (H) GO, and (I) KEGG pathway enrichment analyses of DEPs. (J) Venn diagram illustrating the candidate intersection across three datasets. DEPs, differentially expressed proteins; EV, extracellular vesicle; GO, Gene Ontology; HPA, Human Protein Atlas; KEGG, Kyoto Encyclopedia of Genes and Genomes; LUAD, lung adenocarcinoma; LUSC, lung squamous carcinoma; NTA, nanoparticle tracking analysis; NUH, Nagoya University Hospital; PCA, principal component analysis; TCGA, The Cancer Genome Atlas; TEM, transmission electron microscopy; WB, western blotting.

To prioritise more cancer‐enriched candidates from tissue‐derived EVs, we integrated The Cancer Genome Atlas (TCGA) transcriptomes and identified 14 431 differentially expressed genes (DEGs) (TCGA group; Figure S1A–D). Moreover, we curated a reference set of 2074 genes encoding membrane‐localised proteins from the Human Protein Atlas (HPA) (HPA group). Intersecting the NUH, TCGA, and HPA groups identified 24 DEGs, among which 18 exhibited consistent dysregulation patterns across proteomic and transcriptomic levels, and were enriched in glucose transport, metabolism‐ and hypoxia‐associated pathways (Figures 2J and S1E and F). Analysing 106 matched cancer and non‐cancer tissue pairs from TCGA group, eight genes with cancer‐specific dysregulation were selected (Figure 3A), with GLUT1 (encoded by *SLC2A1*) exhibiting the highest importance score (Figure 3B), the highest expression levels (Figures 3C and S2A), the strongest diagnostic performance (Figures 3D and S2B) and prognostic significance (Figure 3E). Immunohistochemistry in this study also supported GLUT1 enrichment in lung malignancies/lesions (Figure S3A). GLUT1 is overexpressed across diverse malignancies, where its upregulation enhances glucose uptake and drives aerobic glycolysis.^3^, ^4^ Notably, accumulating evidence indicates that GLUT1‐related EVs contribute to metabolic reprogramming and oncogenesis by transferring glycolytic phenotypes to recipient cells, providing a biological rationale for exploring EV‐derived GLUT1 as a biomarker.^5^, ^6^ Through immunofluorescence (HCC827 and A549 cells) and immunogold labelling transmission electron microscopy (cell‐derived EVs), we observed membrane‐localised signals of GLUT1 on both cell and vesicle surfaces (Figure 3F and G).

**FIGURE 3:**
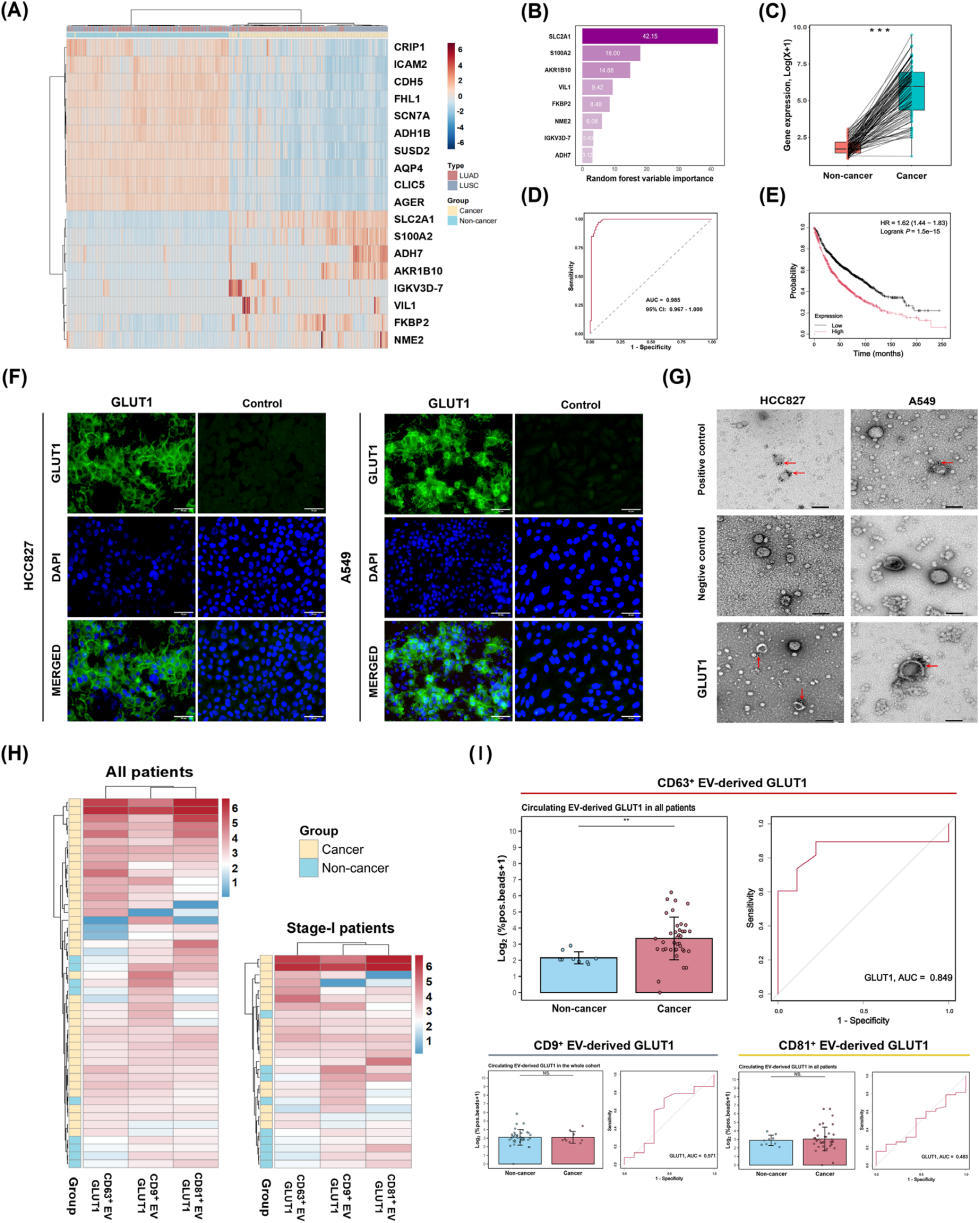
Identification of membrane‐localised, cancer‐specific, circulating EV‐derived biomarker. (A) Hierarchical clustering heatmap of 18 candidates from TCGA paired tissue samples. (B) Variable‐importance bar plot ranking the cancer‐specific candidates. (C) Box plot illustrating expression of *SLC2A1* in different tissues. (D) ROC curve analysis evaluating the diagnostic performance of *SLC2A1*. (E) Kaplan–Meier curve demonstrating overall survival stratified by *SLC2A1*. (F) Immunofluorescence images of HCC827 and A549 cell lines stained for GLUT1 (green) with DAPI nuclear counter‐stain (blue). (G) Immunogold labelling TEM of EVs derived from HCC827 and A549 cell lines. Red arrows indicate EVs positive for the target marker. (H) Hierarchical clustering heatmaps of GLUT1 expression in circulating EVs. (I) Box plots and ROC curves of GLUT1 expression in circulating EVs captured by CD9, CD63, and CD81 in all patients. EVs, extracellular vesicles; GLUT1, glucose transporter 1; LUAD, lung adenocarcinoma; LUSC, lung squamous carcinoma; ROC, receiver operating characteristic; *SLC2A1*, solute carrier family 2 member 1; TCGA, The Cancer Genome Atlas; TEM, transmission electron microscopy.

To overcome the challenge of detecting scarce signals, we applied an ultrasensitive digital EV assay (UDA) incorporating tyramide signal amplification, enabling rapid and sensitive detection of low‐abundance EV‐derived proteins from minimally processed plasma (Figure 1 and Tables S1 and S2).^7^ In the pilot cohort (38 histologically confirmed NSCLC patients and 9 non‐cancer controls), the UDA results demonstrated that CD63^+^ EV‐derived GLUT1 formed a distinct clustering pattern from CD9^+^ and CD81^+^ counterparts (Figure 3H and Table S3). Moreover, CD63^+^ EV‐derived GLUT1 displayed significantly different signals and superior discrimination between cancer and non‐cancer samples, including in stage I patients (Figures 3I and S3B). As reported, EVs bearing CD63 along with one or both of the other tetraspanins probably represent endosome‐derived exosomes, which are thought to play prominent roles in cargo transport and intercellular communication.^8^, ^9^

We also established an overall cohort of 121 NSCLC patients and 36 non‐cancer controls (Table S3). The real‐world overall cohort included IPNs that were radiologically suspicious but could not be definitively classified as malignant or benign using CT alone, reflecting the commonly encountered uncertainty in clinical diagnostic practice. The UDA results revealed that GLUT1 levels were not correlated with sample batch (*r* = .25), and were significantly elevated in cancer samples, including in stage I patients (*p* < .001) (Figure 4A–C). No significant differences in GLUT1 levels were observed across histological subtypes or pathological stages (Figure S3C–E). An exploratory set of inflammatory diseases also indicated lower GLUT1 levels than the cancer group (Figure S3F). The optimal predictor set was selected by LASSO logistic regression with fivefold cross‐validation. For parsimony and clinical interpretability, three variables (GLUT1, CEA, and CYFRA 21‐1) were retained for the final model, which demonstrated diagnostic performance comparable to the full six‐variable model (Figures 4D–H and S4A and B).

**FIGURE 4:**
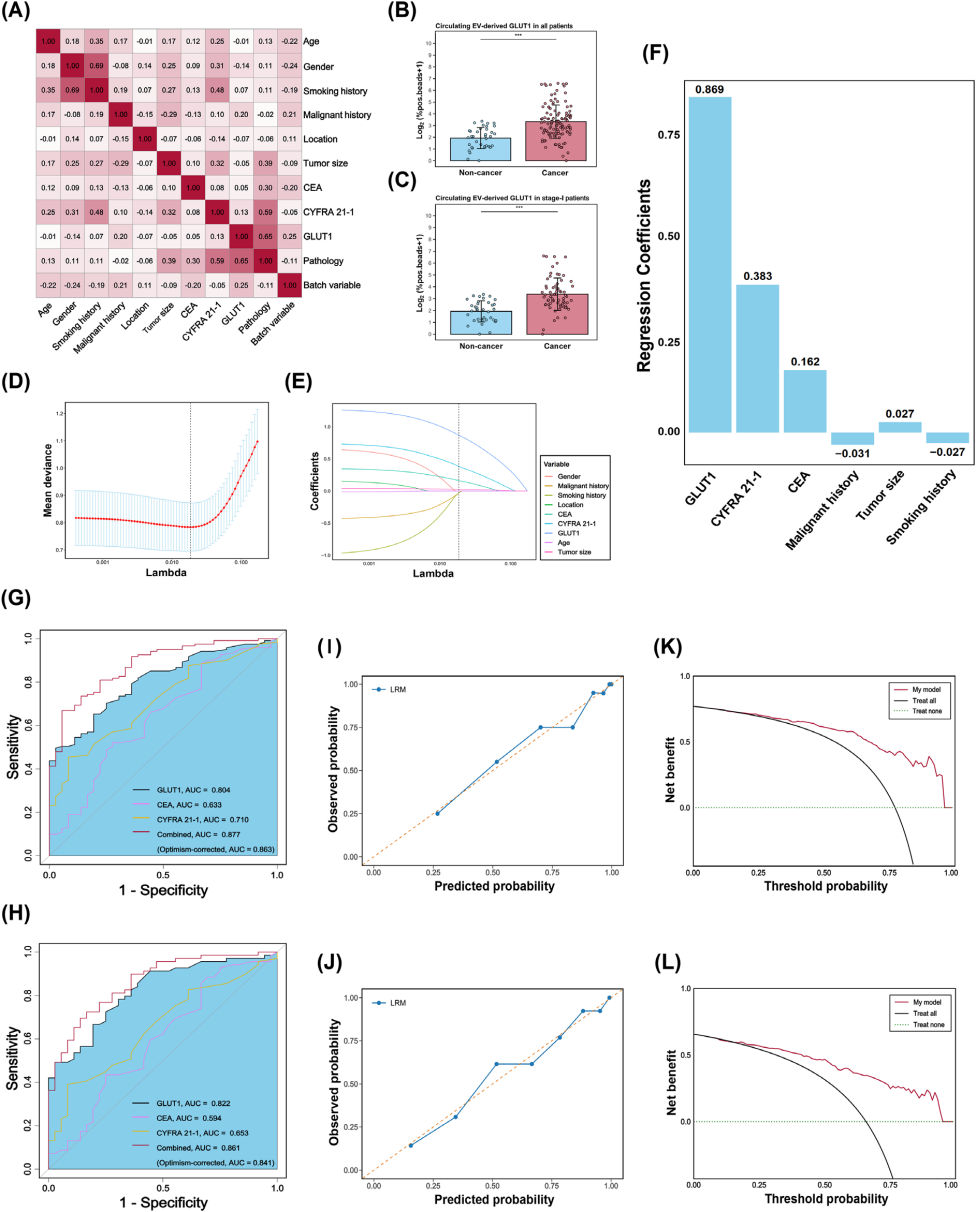
Development of the diagnostic model based on circulating CD63^+^ EV‐derived GLUT1. (A) Correlation heatmap between circulating CD63^+^ EV‐derived GLUT1 and other clinicopathological characteristics. (B) Box plots of circulating CD63^+^ EV‐derived GLUT1 in all patients and (C) in stage I patients. (D) Fivefold cross‐validation curve for optimal λ selection. (E) LASSO coefficient path plot. (F) Regression coefficients of variables selected by the LASSO. (G) ROC curve analysis comparing the diagnostic performance of CD63^+^ EV‐derived GLUT1, CYFRA 21‐1, CEA and the combination in all patients and (H) in stage I patients. (I) Calibration curves of diagnostic model in all patients and (J) in stage I patients. (K) Decision curves of diagnostic model in all patients and (L) in stage I patients. λ, penalty parameter; AUC, area under the curve; CEA, carcinoembryonic antigen; CYFRA 21‐1, cytokeratin 19 fragment; EV, extracellular vesicle; GLUT1, glucose transporter 1; LASSO, least absolute shrinkage and selection operator; LRM, logistic regression model; ROC, receiver operating characteristic.

Specifically, in all patients, CD63^+^ EV‐derived GLUT1 achieved an AUC of  .804 in ROC analysis, outperforming CEA (AUC = .633) and CYFRA 21‐1 (AUC = .710) in discriminating cancer from non‐cancer samples (Figure 4G). Diagnostic performance was improved by the three‐marker panel (AUC = .877; Optimism‐corrected AUC = .863) (Figure 4G). Notably, even in stage I patients, CD63^+^ EV‐derived GLUT1 maintained superior performance (AUC = .822), while the three‐marker panel yielded the improved discrimination (AUC = .861; Optimism‐corrected AUC = .841) (Figure 4H). Favourable calibration curves and low Brier scores ( .113 and  .142) indicated satisfactory overall calibration and accuracy (Figure 4I and J and Table S4). Decision curves showed higher net benefit of the three‐marker panel than treat‐all or treat‐none strategies (Figure 4K and L). Further subgroup analysis of stage IA patients (tumour size < 3 cm) showed consistent findings. CD63^+^ EV‐derived GLUT1 achieved an AUC of  .850 while the three‐marker panel further improved discrimination (AUC = .879; Optimism‐corrected AUC = .855) with acceptable calibration, Brier score, and net benefit (Figure S4C–E and Table S4). Distinguishing malignant from benign nodules remains a major unmet need in thoracic oncology, often leading to unnecessary invasive procedures or delayed diagnosis.^10^ By complementing imaging with molecular information, circulating CD63^+^ EV‐derived GLUT1 may potentially contribute to preoperative malignancy risk assessment and support more informed clinical decision‐making in this diagnostically challenging population.

In conclusion, our findings support a biomarker discovery workflow integrating proteomic profiling of tissue‐derived EVs with ultrasensitive digital qualification in circulating EVs (Figure 1). We identified CD63^+^ EV‐derived GLUT1 as a circulating biomarker for NSCLC, including stage I disease, with promising diagnostic performance that was further enhanced when combined with conventional serum biomarkers. This proof‐of‐concept study indicates the feasibility of translating tissue‐derived EV proteomic signals into non‐invasive circulating biomarkers; however, external validation in larger cohorts and mechanism investigations are critically warranted in subsequent work.

## AUTHOR CONTRIBUTIONS

Conception and design, H.H. and T.K. Data acquisition, H.H., Y.I., Y.N., H.W., H.T., T.R., Y.K., K.N., Y.K., H.U., Y.A., M.K. and E. A‐I. Data analysis and interpretation, H.H., T.K., S.N., T.M., A.T., A.Y. and T. C‐Y. Supervision, T.K. and T. C‐Y. Funding acquisition, T.K. and T. C‐Y. Writing‐original draft, H.H. and T.K. Project administration, T.K. and T. C‐Y. Writing‐review and editing, T.K. S.N., T.M., A.T., A.Y. and T. C‐Y. Final approval of manuscript: all authors.

## CONFLICT OF INTEREST STATEMENT

The authors declare that they have no competing interests.

## FUNDING INFORMATION

This work was supported by JSPS KAKENHI (Grant number: 22K16566 and 24K12009), and the Hori Sciences and Arts Foundation and Nagoya University Hospital Funding for Clinical Research.

## ETHICS STATEMENT

The current study was conducted in accordance with the ethical guidelines and received approval from the Institutional Review Board of Nagoya University Hospital (No.2022‐0107).

## Supporting information



Supporting information

Supporting information

Supporting information

Supporting information

Supporting information

## Data Availability

The data that support the findings of this study are available from the corresponding authors upon reasonable request.
