# Supplementary material for: Tissue‐derived extracellular vesicle profiling identifies GLUT1 enabling ultrasensitive circulating quantification and early detection of non‐small cell lung cancer
Source: Clin Transl Med. 2026 Apr 8;16(4):e70647. doi: 10.1002/ctm2.70647 (PMC13062637; doi:10.1002/ctm2.70647)
Supplement: Supplementary file 1 — Supporting information [file CTM2-16-e70647-s002.docx]

**Letter to the Journal**

**Previous submission number: 3863771**

**Supplementary materials**

**Tissue-derived extracellular vesicle profiling identifies GLUT1 enabling ultrasensitive circulating quantification and early detection of non-small cell lung cancer**

Heng Huang^1†^, Taketo Kato^1†^, Yuichi Abe^2^, Akira Yokoi^3^, Masami Kitagawa^3^, Eri Asano-Inami^3^, Taiki Ryo^1^, Yoshito Imamura^1^, Yuji Nomata^1^, Hirofumi Takenaka^1^, Hiroki Watanabe^1^, Yuta Kawasumi^1^, Keita Nakanishi^1^, Yuka Kadomatsu^1^, Harushi Ueno^1^, Shota Nakamura^1^, Tetsuya Mizuno^1^, Ayumu Taguchi^4^, Toyofumi Fengshi Chen-Yoshikawa^1^

1. Department of Thoracic Surgery, Nagoya University Graduate School of Medicine, Aichi, Japan, 466-8550
2. Immunoproteomics Laboratory, Institute for Glyco-core Research (iGCORE), Gifu University, Gifu, Japan, 501-1193
3. Department of Obstetrics and Gynecology, Nagoya University Graduate School of Medicine, Aichi, Japan, 466-8550
4. Department of Molecular Oncology, Nagoya City University Graduate School of Medical Sciences, Aichi, Japan, 467-8601

^†^ These authors contribute to the work equally.

**Correspondence:** Toyofumi Fengshi Chen-Yoshikawa, Department of Thoracic Surgery, Nagoya University Graduate School of Medicine, 65 Tsurumai-cho, Showa-ku, Nagoya City, Aichi, 466-8550, Japan. Tel: 052-744-2375, E-mail: [yoshikawa.toyofumi.t1@f.mail.nagoya-u.ac.jp](mailto:yoshikawa.toyofumi.t1@f.mail.nagoya-u.ac.jp)

**Methods and materials**

**Clinical sample preparation**

The current study was conducted in accordance with the ethical guidelines and received approval from the Institutional Review Board of Nagoya University Hospital (No.2022-0107). The tissue and blood specimens were collected at Nagoya University Hospital between 2022 and 2025. The inclusion criteria were: pathologically confirmed primary NSCLC or benign lung disease, no preoperative anticancer therapy, and no co-existing malignancies. IPNs represented CT-detected indeterminate nodules that were subsequently confirmed as either malignant or benign by histopathological examination following surgical resection or biopsy. No patient attrition occurred during the study. Whole blood was drawn into purple-top EDTA tubes, kept upright at 4 ℃, and processed within 1 hour of collection. After plasma separation, the samples were transferred to 15 ml tubes and centrifuged at 1500 × g for 10 minutes at 4 ℃. The resulting supernatant was aliquoted and stored at -80 °C until further use.

**Maintenance of cell lines**

The HCC827 and A549 cell lines (derived from a female patient and male patient with adenocarcinoma, respectively) were kindly provided by the Department of Respiratory Medicine, Nagoya University Hospital, and were cultured in Roswell Park Memorial Institute (RPMI) 1640 medium (Thermo Fisher Scientific) supplemented with 10% fetal bovine serum (FBS; Gibco) and 1% penicillin-streptomycin (PS; Gibco).

**Isolation of EVs by density gradient flotation**

Tissue-derived EVs were isolated by ultracentrifugation with density gradient flotation (**Figure 1**).^1^ Tissue specimens were digested enzymatically for 1 hour at 37 ℃ using the Tumor Dissociation Kit (Miltenyi Biotec) in a gentleMACS Octo Dissociator with Heaters. The resulting suspension was centrifuged at 3,000 × g for 15 minutes to remove tissue debris. The supernatant was transferred into Polypropylene Centrifuge Tubes (Beckman Coulter Inc., USA) and mixed with filtered iodixanol solution to a final density of ~1.163-1.189 g/ml. A 2 ml layer of 1.134 g/ml iodixanol solution was carefully overlaid to establish a discontinuous density gradient. Ultracentrifugation was performed at 65,000 × g for more than 12 hours at 4°C using an Optima XE-100 ultracentrifuge with an SW 41 Ti Swinging-Bucket rotor (Beckman Coulter Inc., USA). A 500 μl fraction of the supernatant was collected and filtered through 0.22 μm membrane filters for subsequent characterization.

For the isolation of cell-derived EVs, the culture medium was replaced with Advanced RPMI 1640 medium (Thermo Fisher Scientific) supplemented with 1% EV-depleted FBS at ~70% cell confluence. Then the conditioned media were collected and centrifuged at 2,000 × g for 10 minutes, followed by filtration through a Vacuum Filter/Storage Bottle System (150 ml, 0.22 [μ](https://zh.wiktionary.org/wiki/%CE%BC)m diameter; Corning). The filtered medium was then aliquoted into Amicon^®^ Ultra-15ml Centrifugal Filters and centrifuged at ~2,600 × g. Ultracentrifugation was performed using the same procedure as for tissue-derived EVs. After that, 1 ml of supernatant was transferred to Amicon^®^ Ultra-2ml Centrifugal Filters, and centrifuged at ~2,600 × g until the medium was concentrated to about 150 μl for subsequent characterization.

**Characterization of EVs**

***Nanoparticle tracking analysis (NTA)***

Size distribution, particle concentration, and mode size of EVs were quantitatively analyzed using NTA with the NanoSight NS300 system (Malvern Panalytical Ltd., UK). EV samples were diluted 1:100 in phosphate-buffered saline (PBS). Triplicate measurements were performed for each sample, with individual recording duration of 60 seconds to ensure statistical reliability.

***Transmission electron microscopy (TEM)***

For morphological assessment, 10 μl of EV suspension was loaded onto carbon-coated copper grids (Nisshin EM, Japan), and negatively stained with 1% aqueous uranyl acetate, followed by air dry at room temperature. For immunogold labeling, 5 μl of EV suspension was loaded onto carbon-coated grids and processed similarly, followed by blocking with 1% bovine serum albumin (BSA) for 1 hour at room temperature. Grids were then incubated with a primary antibody (Anti-Glucose Transporter GLUT1 antibody, Abcam, ab115730, rabbit, dilution 1:50) for 1 hour at room temperature. After PBS washes, grids were incubated with a 10-nm gold-labeled secondary antibody [anti-IgG (H+L) Rabbit Goat-Poly Gold 10nm, EMGAR 10, CRL, Cardiff, UK, dilution 1:50] for another hour. After PBS washes, the grids were fixed with 1% glutaraldehyde for 10 minutes, rinsed with distilled water, stained with 1% uranyl acetate, blotted with filter paper, and air-dried. Imaging was performed using a JEM-1400Plus TEM (JEOL Ltd., Japan).

***Western blotting (WB)***

EV-derived protein quantification was performed using the Qubit™ Protein and Protein Broad Range Assay Kits (Thermo Fisher Scientific) with the Qubit 4.0 Fluorometer (Invitrogen Co., USA). EV samples were subjected to precast polyacrylamide gel electrophoresis at a constant current of 42 mA (ATTO Corporation). Then proteins were transferred onto polyvinylidene difluoride (PVDF) membranes at 24 V for 15 minutes using a semi-dry transfer system (ATTO Corporation). Membranes were blocked with 5% (w/v) skim milk (MORINAGA & CO., LTD., Japan) in Tris-buffered saline with Tween 20 (TBST, ATTO Corporation) for 1 hour at room temperature, followed by 3 TBST washes and overnight incubation at 4°C with primary antibodies: rabbit monoclonal anti-TSG101 (Abcam, ab125011, dilution 1:500), rabbit monoclonal anti-CD9 (Cell Signaling Technology, D8O1A, dilution 1:1000), and rabbit monoclonal anti-calnexin (Cell Signaling Technology, C5C9, dilution 1:1000). After 3 TBST washes, membranes were incubated for 2 hours at room temperature with HRP-linked goat anti-rabbit IgG secondary antibody (Cell Signaling Technology, 7074S; dilution 1:2000). After 3 TBST washes, protein bands were visualized using the ImageQuant LAS 4010 system (GE Healthcare, IL, USA).

**Mass spectrometry (MS)-based proteomic profiling**

EVs isolated from tissues and cell lines were lysed in 1% sodium dodecyl sulfate (SDS; FUJIFILM Wako) supplemented with a cOmplete™ protease inhibitor cocktail (Roche), followed by boiling at 100 °C for 5 minutes and sonication at 4 °C for 15 minutes. Ten micrograms of total protein were subjected to reduction with dithiothreitol (FUJIFILM Wako) and alkylation with iodoacetamide (Nacalai Tesque, Inc.). Proteins were precipitated onto carboxylate-modified magnetic particles (Sera-Mag SpeedBeads; Cytiva) in 50% ethanol (FUJIFILM Wako) and washed three times with 80% ethanol to remove contaminants. The resulting protein aggregates were resuspended in 50 mM ammonium bicarbonate (FUJIFILM Wako) containing trypsin (Roche) at a protein-to-enzyme ratio of 50:1 and digested overnight at 37 °C. Digestion was terminated by the addition of 1% trifluoroacetic acid (FUJIFILM Wako).

Peptides equivalent to 8 µg of protein were desalted using C18 StageTips. Desalted peptides were labeled with Tandem Mass Tag (TMT) 10-plex reagents (Thermo Fisher Scientific) according to the manufacturer’s instructions. A total of 24 µg of the TMT-labeled peptide mixture (corresponding to 80 µg of protein) was fractionated into seven fractions on a C18/SCX StageTip using via pH and salt-based elution, followed by vacuum evaporation.

Each fraction was analyzed on a Q Exactive mass spectrometer coupled with an UltiMate 3000 nanoLC system (Thermo Fisher Scientific, USA), equipped with a nano-electrospray ionization (nano-ESI) source (AMR, Japan) and a 150 mm × 75 µm inner diameter capillary column (Nikkyo Technos, Japan). Low-pH reverse-phase liquid chromatography (RPLC) was performed at a flow rate of 300 nl/min using a linear gradient elution starting from 5% to 40% solvent B (95% acetonitrile with 0.1% formic acid) over 100 minutes. Solvent A consisted of 2% acetonitrile with 0.1% formic acid. MS1 scans were acquired in the mass-to-charge (m/z) range of 400-1600. The top 20 most intense precursor ions were selected for higher-energy collisional dissociation (HCD) and MS/MS analysis in a data-dependent acquisition (DDA) mode.

Protein identification and quantification were performed using MaxQuant software (version 1.6.7.0; <http://maxquant.org>) with the integrated Andromeda search engine. MS/MS spectra were searched against the UniProt human reference proteome database (release 2019_04; https://www.uniprot.org/), supplemented with 262 common contaminant proteins. Trypsin/P was specified as the digestion enzyme, allowing up to two missed cleavages. Carbamidomethylation of cysteine residues and TMT labeling of lysine residues and peptide N-termini were set as fixed modifications. Oxidation of methionine and deamidation of asparagine and glutamine were considered variable modifications. The false discovery rate (FDR) was controlled to be < 1% at the levels of protein groups, peptide groups, and peptide-spectrum matches (PSMs). Quantitative values for each protein group were median-normalized and log2-transformed prior to statistical analysis. Differentially expressed proteins (DEPs) of tissue-derived EVs were initially screened using Student’s t-test with relaxed thresholds (absolute fold change [FC] > 1.2 or < 0.83) to avoid loss of biologically relevant low-abundance EV proteins.^2, 3^ To enhance robustness and prioritize cancer-specific signals, these candidates were subsequently cross-filtered using transcriptomic data.

**Transcriptomic profiling from public database**

Publicly available transcriptomic data and matched clinical information of patients with NSCLC were obtained from The Cancer Genome Atlas (TCGA) via Genome Data General Database (GDC) data portal and analyzed using bioinformatic methods. A custom Wilcoxon test-based function was applied to identify differentially expressed genes (DEGs) between cancer and non-cancer tissues, using criteria of |log(fold change)| ≥ 1 and FDR < 0.05. A gene set encoding plasma membrane-localized proteins was retrieved from the Human Protein Atlas (HPA) database, and served as a reference to screen the candidate target DEGs.

**Immunofluorescence for protein localization**

HCC827 and A549 cells were seeded onto 4-well chamber slides (WATSON BIO LAB) and cultured to ~70% confluence. Cells were washed twice with PBS, fixed with 10% formalin for 15 minutes, and then permeabilized with 0.1% Triton X-100 (Sigma) in PBS for 5 minutes, followed by two additional washes. Non-specific binding sites were blocked with 0.5% BSA for 30 minutes at room temperature. Samples were incubated overnight at 4°C with a primary antibody (Anti-Glucose Transporter GLUT1 antibody, Abcam, ab115730, rabbit, dilution 1:100) diluted in 0.5% BSA. After two PBS washes, cells were incubated with a secondary antibody (Goat Anti-Rabbit IgG H&L, Alexa Fluor 488-conjugated, Abcam, ab150077, dilution 1:500) for 1 hour at room temperature in the dark, followed by two washes. Slides were briefly air-dried, and nuclei were counterstained with one drop of DAPI Fluoromount-G (Cosmo Bio). Coverslips were mounted onto glass slides and sealed by nail polish. Fluorescence images were captured using a BZ-9000 fluorescence microscope system at 40× magnification (KEYENCE Corporation, Japan). Merged images of DAPI (blue) and GLUT1 (green) channels were generated to assess the subcellular localization of GLUT1.

**Ultrasensitive digital EV assay (UDA)**

Experimental protocols of the UDA, including reagent specification, incubation time and concentration, were outlined in **Figure 1 and Table S1,2**. EVs were immunocaptured using magnetic beads coated with anti-tetraspanin antibodies. Membrane-associated target signals were detected and amplified by biotinylated tyramide under the HRP catalysis. Beads readouts were acquired using a BD FACS Canto™ II Flow Cytometer with the following settings: forward scatter (530 V), side scatter (390 V), and pacific blue (530 V). Buffer-only control and buffer with reagents control were applied to quantify the contribution of background noise.^4^ A quadrant gate was used to delineate the positive bead populations. All data were collected as the percentage of positive beads out of 10,000 total events.

**Immunohistochemistry (IHC)**

Paraffin-embedded lung tissue sections were deparaffinized in Hemo-De (Falma Co., LTD.), and rehydrated through gradient ethanol solution. Heat-induced antigen retrieval was performed in citrate buffer (pH 6.0, Sigma) jin a pressure-based chamber (about 70 kPa / 115 ℃), followed by cooling to room temperature. Endogenous peroxidase activity was blocked with hydrogen peroxide (FUJIFILM Wako) followed by TBST washes. Non-specific binding was blocked with Blocking One Histo (Nacalai Tesque, Inc.), after which sections were incubated with the primary antibody (Anti-Glucose Transporter GLUT1 antibody, Abcam, ab115730, rabbit, dilution 1:2000) for 2 hours at room temperature. After TBST washes, a polymer-based HRP detection reagent (SignalStain[R] Boost IHC Det. [HRP, Rab], Cell Signaling Technology) was applied at room temperature. Signal was developed with 3,3’-diaminobenzidine (DAB, MBL Life Science), while nuclear staining was performed with hematoxylin (Sigma). Sections were then dehydrated through gradient ethanol solution, cleared in Hemo-De, and mounted with DPX Mountant (Sigma) under coverslips. For each section, multiple fields were sampled from both the lesional tissue and the adjacent normal lung tissue, and the positive area was quantified using a BZ-X800 microscope system (KEYENCE Corporation, Japan).

**Statistical analysis**

All statistical analyses and data visualizations were performed using R software (R Foundation for Statistical Computing, v4.3.3). Continuous variables were presented as mean ± standard deviation (SD) or median with interquartile range (IQR), and compared using Student’s *t*-test or the Mann-Whitney *U* test, as appropriate. Categorical variables were reported as percentages. Unsupervised hierarchical clustering heatmaps were generated using the *heatmap* package (v1.0.12). Principal component analysis (PCA) plots, volcano plots, and box plots were created using the *ggplot2* package (v3.5.1). Functional enrichment analyses were conducted using the *clusterProfiler* package (v4.10.1). Variable importance was estimated using the *randomForest* package (v4.7-1.2), representing the relative contribution of each variable to the model performance. Least absolute shrinkage and selection operator (LASSO) regression with 5-fold cross-validation was performed for feature selection using the *glmnet* package (v4.1-8). Logistic regression model was fitted, and internal validation was performed via bootstrap resampling with optimism correction using the *rms* package (v6.8-1). Receiver operating characteristic (ROC) curves and area under the curve (AUC) values were computed using the *pROC* package (v1.18.5). Calibration curves were plotted using the *ggplot2* package (version 3.5.1) to assess the agreement between predicted and observed probabilities. Model accuracy was further evaluated using Brier scores, computed through 1,000 bootstrap iterations; lower scores indicate better performance. Decision curve analysis (DCA) was conducted using the *rmda* package to assess the clinical net benefit of the diagnostic model. A *P* value < 0.05 was considered statistically significant.

**References**

1. Crescitelli R, Lasser C, Lotvall J. Isolation and characterization of extracellular vesicle subpopulations from tissues. *Nat Protoc*. 2021; 16(3): 1548–1580.

2. Zhong ME, Chen Y, Xiao Y, et al. Serum extracellular vesicles contain SPARC and LRG1 as biomarkers of colon cancer and differ by tumour primary location. *EBioMedicine*. 2019; 50: 211–223.

3. Yang J, Lv Z, Liu L, et al. Peripheral blood neutrophil proteomic profiling with transcriptomic data integration reveals biomarkers for tuberculosis infection diagnosis. *EBioMedicine*. 2025; 120: 105945.

4. Welsh JA, Arkesteijn GJA, Bremer M, et al. A compendium of single extracellular vesicle flow cytometry. *J Extracell Vesicles*. 2023; 12(2): e12299.

**Figure legends**

**Figure S1. Identification and functional annotation of candidates from the TCGA and the intersection groups.** (**A**) Volcano plot, (**B**) hierarchical clustering heatmap, (**C**) GO, and (**D**) KEGG pathway enrichment analyses of DEGs from the TCGA group. (**E**) GO, and (**F**) KEGG pathway enrichment analyses of the 18 candidates with consistent dysregulation patterns across proteomic and transcriptomic levels. **Abbreviations**: DEPs, differentially expressed genes; TCGA, The Cancer Genome Atlas; GO, Gene Ontology; KEGG, Kyoto Encyclopedia of Genes and Genomes.

**Figure S2. Gene expression and diagnostic performance of the other seven candidates from the TCGA group.** (**A**) Box plots, and (**B**) ROC curves of the other seven candidates from the TCGA group. **Abbreviations**: TCGA, The Cancer Genome Atlas; ROC, receiver operating characteristic; AUC, area under the curve; CI, confidence interval.

^*^ All analyses in panels A and B were performed on 106 paired tumor-normal tissue samples from the TCGA group.

**Figure S3. Tissue-level and circulating CD63^+^** **EV-derived GLUT1 across clinicopathological characteristics in NSCLC.** (**A**) IHC staining and quantification of GLUT1 in lesional and adjacent normal lung tissues. (**B**) Box plots and ROC curves of GLUT1 expression in circulating EVs captured by CD9, CD63, and CD81 in stage-I patients. (**C-D**) Box plots illustrating CD63⁺ EV-derived GLUT1 expression stratified by histological subtype and (**E**) pathological stage. (**F**) Box plot of circulating CD63^+^ EV-derived GLUT1 in different lung diseases. **Abbreviations**: EV, extracellular vesicle; vesicle; GLUT1, glucose transporter 1; NSCLC, non-small cell lung cancer; IHC, immunohistochemistry; ROC, receiver operating characteristic; AUC, area under the curve; LUAD, lung adenocarcinoma; LUSC, lung squamous carcinoma.

**Figure S4. Diagnostic performance of six- and three- marker panels in NSCLC.** (**A**) ROC curve analyses comparing the diagnostic performance of six-marker panel in all patients, and (**B**) in stage-I patients. (**C**) ROC curve analysis comparing the diagnostic performance of three-marker panel in stage-IA patients, with the (**D**) calibration curve and (**E**) decision curve. **Abbreviations**: NSCLC, non-small cell lung cancer; ROC, receiver operating characteristic; AUC, area under the curve; GLUT1, glucose transporter 1; CEA, carcinoembryonic antigen; CYFRA 21-1, cytokeratin 19 fragment; LRM, logistic regression model.

**Tables**

**Table S1. Experimental protocols of the ultrasensitive digital EV assay.**

| **Step** | **Reagent / Procedure** | **Time (min)** | **Volume (μl)** | **Concentration** | **Buffer** |
| --- | --- | --- | --- | --- | --- |
| 1 | Magnetic beads ^#^ | 30 | 100 | 2 μl/well | 2% BSA |
| 2 | Washing × 4 |  | 100 |  | PBS + 0.1% Tween-20 |
| 3 | Plasma sample | 60 | 100 | 5-10 μl | 2% BSA |
| 4 | Washing × 4 |  | 100 |  | PBS + 0.1% Tween-20 |
| 5 | Detection antibody | 60 | 50 | 1 μg/ml | 2% BSA |
| 6 | Washing × 4 |  | 100 |  | PBS + 0.1% Tween-20 |
| 7 | Streptavidin-HRP | 30 | 100 | 1:200 | 2% BSA |
| 8 | Washing × 4 |  | 100 |  | PBS + 0.1% Tween-20 |
| 9 | Biotinylated tyramide | 10 | 100 | 35 μg/ml | 0.1 mol/L Borate buffer (pH 8.5)  + 0.003% H_2_O_2_ |
| 10 | Washing × 4 |  | 100 |  | PBS + 0.1% Tween-20 |
| 11 | Fluorescence-labeled streptavidin | 30 | 50 | 1:1000 | 2% BSA |
| 12 | Washing × 4 |  | 100 |  | PBS + 0.1% Tween-20 |
| 13 | FACS test |  | 250 |  |  |

^#^ Magnetic beads were individually coated with anti-tetraspanin antibodies as needed. Specifically, anti-CD9, anti-CD63, and anti-CD81 antibodies were used in the pilot cohort, whereas only anti-CD63 antibody was applied in the overall cohort.

^*^ Beads are collected using a 96-well plate magnet in each washing step. Each incubation is performed on a plate shaker to prevent the beads deposition. Washing times could be adjusted according to experimental requirement. **Abbreviations**: BSA, bovine serum albumin; PBS, phosphate buffered saline; HRP, horseradish peroxidase; H_2_O_2_, hydrogen peroxide solution; FACS, fluorescence-activated cell sorting.

**Table S2. List of reagents used in the ultrasensitive digital EV assay.**

| **Product name** | **Company** | **Catalog number** | **Specification** | **Concentration used** |
| --- | --- | --- | --- | --- |
| CD9 (D8O1A) Rabbit mAb | Cell Signaling | 13174S | 84 μg/ml, 100 μl | 84 μg/ml, 100 μl |
| CD63 (D4I1X) Rabbit mAb | Cell Signaling | 55051S | 11 μg/ml, 100 μl | 11 μg/ml, 100 μl |
| CD81 (D3N2D) Rabbit mAb | Cell Signaling | 56039S | 50 μg/ml, 100 μl | 50 μg/ml, 100 μl |
| Dynabeads™ Antibody Coupling Kit | Thermo Fisher | 14311D | 60 mg | 5 mg/500 μl |
| BSA | Sigma-Aldrich | A7030 | 100 g | 2% |
| PBS | Thermo Fisher | 10010-023 | 1X/500 ml | - |
| Tween-20 | Sigma-Aldrich | P1379 | 100ml | 0.1% |
| Zeba™ Spin Desalting Columns | Thermo Fisher | 89882 | 0.5 ml | - |
| EZ-Link™ Sulfo-NHS-LC-Biotin | Thermo Fisher | A39257 | 1 mg | 1 mg/180 μl |
| Anti-Glucose Transporter GLUT1 antibody | Abcam | ab115730 | 0.179 mg/ml, 100 μl | 1 μg/ml |
| Streptavidin-HRP | R&D Systems | DY998 | 1 ml, 1:200 | 1:200 |
| Biotinyl tyramide | Sigma-Aldrich | SML2135-50MG | 50 mg | 35 μg/ml |
| Boric acid | Sigma-Aldrich | B6768-500G | 500 g | 0.1 mol/L |
| Hydrogen peroxide solution | FUJIFILM Wako | 081-04215 | 30%, 500 ml | 0.003% |
| Brilliant violet 421 streptavidin | BioLegend | 405225 | 0.5 mg/ml, 200 μl | 1:1000 |

^*^ **Abbreviations**: BSA, bovine serum albumin; PBS, phosphate buffered saline; GLUT1, glucose transporter 1; mAb, monoclonal antibody; HRP, horseradish peroxidase; TSA, tyramide signal amplification; DMSO, dimethyl sulfoxide.

**Table S3. The baseline characteristics of patients included in the study.**

| **Characteristics** | **Pilot cohort**  **N (%)** | **Overall cohort**  **N (%)** |
| --- | --- | --- |
| Total cases | 47 (100) | 157 (100) |
| Age | 70.00 ± 9.71 | 67.50 ± 9.61 |
| Gender |  |  |
| Male | 38 (80.85) | 113 (71.97) |
| Female | 9 (19.15) | 44 (28.03) |
| Smoking history |  |  |
| Yes | 38 (80.85) | 116 (73.89) |
| No | 9 (19.15) | 41 (26.11) |
| Malignant history |  |  |
| Yes | 8 (17.02) | 38 (24.20) |
| No | 39 (82.98) | 119 (75.80) |
| Location |  |  |
| Upper lobe | 21 (44.68) | 78 (49.68) |
| Other lobe | 26 (55.32) | 79 (50.32) |
| Tumor size | 32.66 ± 22.42 | 30.85 ± 18.66 |
| Pathological diagnosis |  |  |
| Cancer | 38 (80.85) | 121 (77.07) |
| Non-cancer | 9 (19.15) | 36 (22.93) |
| Subtype ^*^ |  |  |
| LUAD | 24 (63.16) | 68 (56.20) |
| LUSC | 13 (34.21) | 45 (37.19) |
| Other | 1 (2.63) | 8 (6.61) |
| Stage ^*^ |  |  |
| I | 18 (47.37) | 69 (57.02) |
| II/III/IV | 20 (52.63) | 52 (42.98) |

^*^ Subtype and stage characteristics were collected from the patients with NSCLC, and were not applicable to the non-cancer patients. **Abbreviations:** NSCLC, non-small cell lung cancer; LUAD, lung adenocarcinoma; LUSC, lung squamous carcinoma.

**Table S4. The diagnostic performance of three-marker panel (CD63^+^ EV-derived GLUT1 + CEA + CYFRA 21-1) in NSCLC.**

| **Models** | **AUC** | **Sensitivity** | **Specificity** | **Brier score** |
| --- | --- | --- | --- | --- |
| **Whole cohort** | 0.877 (0.819-0.936) | 0.669 (0.578-0.752) | 0.944 (0.813-0.993) | 0.113 (0.084-0.145) |
| **Stage-I cohort** | 0.861 (0.791-0.932) | 0.725 (0.604-0.825) | 0.833 (0.672-0.936) | 0.142 (0.105-0.185) |
| **Stage-IA cohort** | 0.879 (0.806-0.953) | 0.619 (0.456-0.764) | 0.972 (0.855-0.999) | 0.144 (0.102-0.192) |

^*^ EV, extracellular vesicle; GLUT1, glucose transporter 1; CEA, carcinoembryonic antigen; CYFRA 21-1, cytokeratin 19 fragment; NSCLC, non-small cell lung cancer; AUC, area under the curve.
