# Supplementary figures and images for: Tissue‐derived extracellular vesicle profiling identifies GLUT1 enabling ultrasensitive circulating quantification and early detection of non‐small cell lung cancer
Source: Clin Transl Med. 2026 Apr 8;16(4):e70647. doi: 10.1002/ctm2.70647 (PMC13062637; doi:10.1002/ctm2.70647)

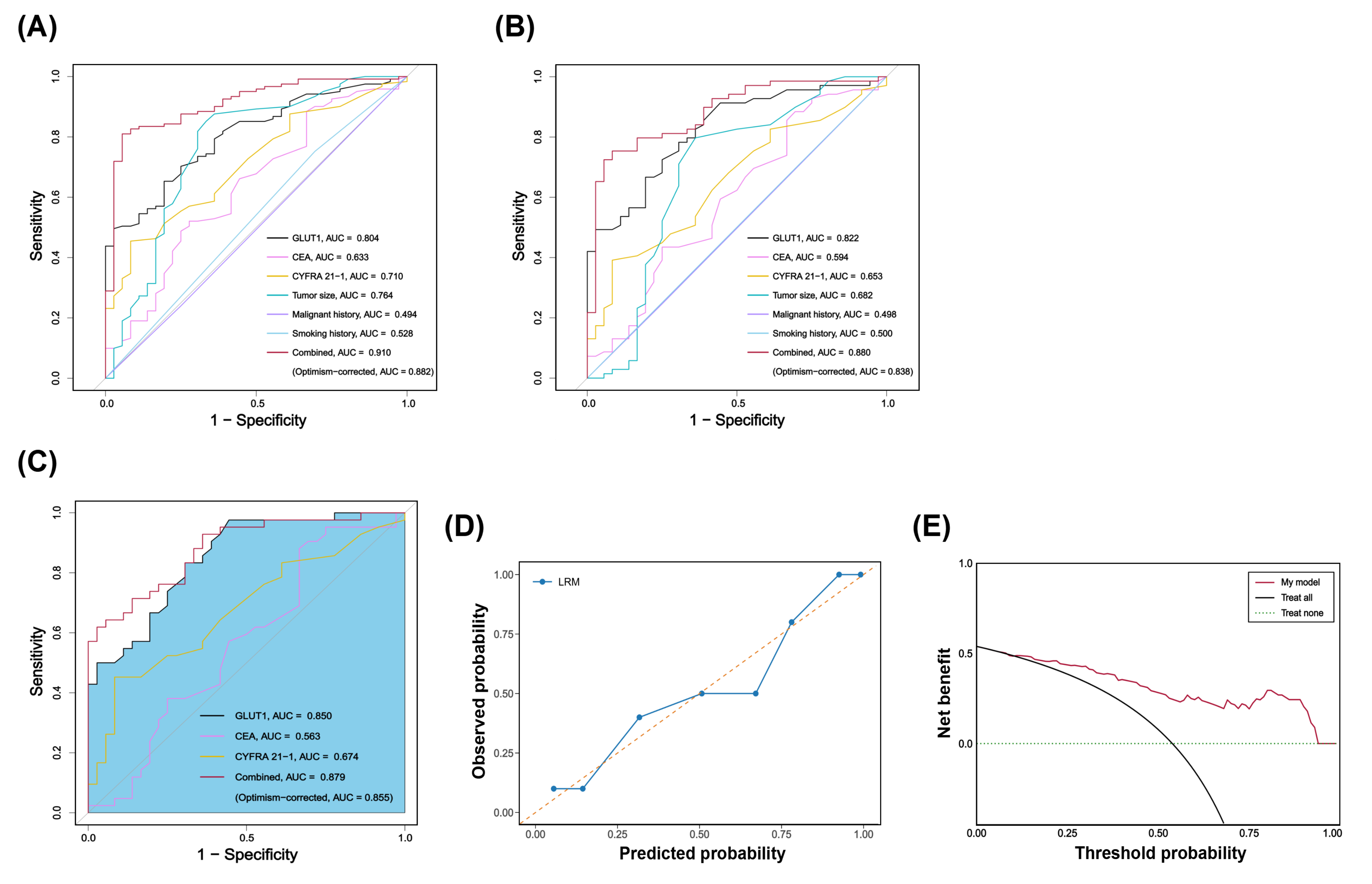

Supplement: Supplementary file 5 — Supporting information [file CTM2-16-e70647-s001.png]
